# Minimum Effective Dose of DPP-4 Inhibitors for Treating Early Stages of Diabetic Retinopathy in an Experimental Model

**DOI:** 10.3390/biomedicines10020465

**Published:** 2022-02-16

**Authors:** Patricia Bogdanov, Hugo Ramos, Marta Valeri, Anna Deàs-Just, Jordi Huerta, Rafael Simó, Cristina Hernández

**Affiliations:** 1 Diabetes and Metabolism Research Unit, Vall d’Hebron Research Institute, 08035 Barcelona, Spain; patricia.bogdanov@vhir.org (P.B.); hugo.ramos@vhir.org (H.R.); anna.deas@vhir.org (A.D.-J.); jordi.huerta@vhir.org (J.H.); 2 Centro de Investigación Biomédica en Red de Diabetes y Enfermedades Metabólicas Asociadas (CIBERDEM), Instituto de Salud Carlos III (ICSIII), 28029 Madrid, Spain; 3 Unit of High Technology, Vall d’Hebron Research Institute, 08035 Barcelona, Spain; marta.valeri@vhir.org; 4 Department of Medicine, Universitat Autònoma de Barcelona, 08193 Barcelona, Spain

**Keywords:** diabetic retinopathy, dipeptidyl peptidase-4 inhibitors, sitagliptin, saxagliptin, neurovascular unit, retinal neurodegeneration, experimental diabetes, db/db mice

## Abstract

The neurovascular unit (NVU) plays an essential role in the development of diabetic retinopathy (DR). We previously reported that the topical administration (eye drops) of sitagliptin and saxagliptin, two dipeptidyl peptidase-4 inhibitors (DPP-4i), prevents retinal neurodegeneration and vascular leakage in db/db mice. The aim of the present study is to evaluate the minimum effective dose of the topical administration of these DPP-4i. For this purpose, sitagliptin and saxagliptin were tested at different concentrations (sitagliptin: 1 mg/mL, 5 and 10 mg/mL, twice per day; saxagliptin: 1 and 10 mg/mL, once or twice per day) in db/db mice. As end points of efficacy, the hallmarks of NVU impairment were evaluated: reactive gliosis, neural apoptosis, and vascular leakage. These parameters were assessed by immunohistochemistry, cell counting, and the Evans blue method, respectively. Our results demonstrated that the minimum effective dose is 5 mg/mL twice per day for sitagliptin, and 10 mg/mL twice per day for saxagliptin. In conclusion, this study provides useful results for the design of future preclinical regulatory studies and for planning clinical trials.

## 1. Introduction

Diabetic retinopathy (DR) is the leading cause of visual impairment and preventable blindness and represents a significant socioeconomic cost for healthcare systems worldwide [1,2]. The current treatments for DR, such as laser photocoagulation, intra-vitreal injections of corticosteroids, intra-vitreal injections of anti-vascular endothelial growth factor (VEGF) agents, and vitreoretinal surgery, are expensive, have a significant number of secondary effects, and all of them are only used in the advanced stages of DR [3]. At present, treatment of the classic risk factors of DR (i.e., hyperglycemia and hypertension) is the only therapeutic strategy against the early stages, giving rise to an unmet medical need that has to be filled [4].

Since neurodegeneration is a well-established event in the pathogenesis of DR [5], several neuroprotective strategies have emerged as successful treatments in the early stages of experimental models of DR. One such strategy is based on the replacement of neuroprotective factors, such as pigment epithelium-derived factor (PEDF), somatostatin, and glucagon-like peptide 1 (GLP-1), which are downregulated in the diabetic retina [6]. GLP-1 and GLP-1R agonists (GLP-1RAs) have been the most studied, and the main mechanisms underlying their neuroprotective properties are: (1) anti-inflammatory action; (2) the inhibition of excitotoxicity and neuron death by reducing glutamate accumulation in the extracellular space; (3) antiapoptotic action by increasing antiapoptotic mediators (i.e., FasL, capase 8, P53/p-P53, Bax) and downregulating survival pathways (Bcl-xL, Bcl-2); (4) antioxidant properties; and (5) a neurogenic effect by restoring retinal neuron cells and reactive gliosis. In addition to neuroprotective action, GLP-1RAs also have microvascular protection by several mechanisms, including the prevention of the downregulation of tight junction proteins, and the inhibition of the overexpression of VEGF and PlGF, and pro-inflammatory cytokines induced by diabetes [6].

The replacement treatment with these factors seems a reasonable approach for preventing retinal neurodegeneration and has been successfully used in experimental models [7,8,9,10,11,12]. However, the systemic administration of these neuropeptides can hardly reach the retina at pharmacological concentrations and, by contrast, could have systemic adverse effects [4]. On the other hand, when the early stages of DR are the therapeutic target, it would be inconceivable to recommend an aggressive treatment such as intravitreal injections [4]. For all these reasons, the ocular topical route has emerged as an effective therapeutic approach to deliver these neuroprotective factors into the diabetic retina.

We have previously demonstrated that the topical administration of sitagliptin and saxagliptin (two DPP-4 inhibitors) has a powerful action in preventing both neurodegeneration and vascular leakage, two hallmarks of neurovascular unit impairment that occurs in the early stages of DR [13]. As occurs in the systemic circulation, the main mechanism of action of topical (eye drops) DPP-4 inhibitors relies on its capacity of inhibiting GLP-1 degradation. This effect favors the enhancement of GLP-1 content in the neuroretina, thus accounting for the dual action (neuroprotective and vasculotropic). In addition, sitagliptin and saxagliptin were able to significantly increase the levels of exchange protein activated by cAMPc (EPAC-1), which plays an important role in the maintenance of the endothelial barrier and neuronal functions. However, the simultaneous activation of other mechanisms unrelated to GLP-1R activation cannot be ruled out.

Given the reported powerful effect of eye drops of sitagliptin and saxagliptin (at doses of 50 mg/mL and 31.5 mg/mL, respectively) in preventing neurodegeneration and vascular leakage [13], we wanted to determine whether a lower dose could also be effective. Therefore, the aim of the present study was to determine the minimum effective dose of sitagliptin and saxagliptin in eye drops for preclinical regulatory purposes and planning future clinical trials.

## 2. Materials and Methods

### 2.1. Experimental Design

A total of 63 diabetic male db/db (BKS.Cg-Dock7m +/+ Leprdb/J) mice and 14 non-diabetic mice db/+; (BKS.Cg-Dock7m + Leprdb/+), aged 8 weeks, were purchased (Charles River Laboratories, Calco, Italy) for the study. Db/db mice carry a mutated leptin receptor that leads to obesity-induced type 2 diabetes. The animals had free access to ad libitum food (ENVIGO Global Diet Complete Feed for Rodents, Mucedola, Milan, Italy) and filtered water. They were maintained at all times at 20 °C temperature and 60% humidity. In order to minimize variability, the animals were randomly housed (block randomization) in groups of 4 mice per cage. Each cage held absorbent bedding and nesting material (BioFresh Performance Bedding 1/800 Pelleted Cellulose, Absorption Corp, Ferndale, WA, USA).

### 2.2. Interventional Study

At the age of 10 weeks, sitagliptin 1 mg/mL (twice daily, *n* = 7), sitagliptin 5 mg/mL (twice daily, *n* = 7), sitagliptin 10 mg/mL (twice daily, *n* = 7), saxagliptin 1 mg/mL (*n* = 14) (once and twice daily, *n* = 7 in each case), saxagliptin 10 mg/mL (*n* = 14) (once and twice daily, *n* = 7 in each case) and vehicle eye drops (*n* = 14) were randomly administered directly onto the superior corneal surface of each db/db eye using a micropipette (one drop: 5 µL). Sitagliptin (1, 5 or 10 mg/mL), saxagliptin (1 or 10 mg/mL) or vehicle (5 µL phosphate-buffered saline (PBS), pH 7.4) was administrated for 15 days in each eye. On the last day (12 weeks of age), one drop of sitagliptin, saxagliptin or vehicle was administered to each eye 1 h before euthanasia. Fourteen non-diabetic mice (db/+) matched by age served as the control group.

This study was approved by the Animal Care and Use Committee of VHIR (Vall d’Hebron Research Institute, Barcelona, Spain). All the experiments were performed in accordance with the tenets of the European Community (86/609/CEE) and the Association for Research in Vision and Ophthalmology (ARVO).

### 2.3. Retinal Tissue Processing

On day 15, 4 mice of each experimental group were transcardiacally perfused with p-formaldehyde 4% (sc-281692, Santa Cruz Biotechnology, Dallas, TX, USA), and the eyes were immediately enucleated, fixed in paraformaldehyde 4% for 5 hours, and embedded in paraffin blocks. Previously, each animal was intraperitoneally injected with 200 µL of anaesthesia prepared with a mix containing 1 mL ketamine (GmbH, Hameln, Germany) and 0.3 mL xylazine (Laboratorios Calier S.A., Barcelona, Spain).

### 2.4. Retinal Vascular Permeability by Evans Blue Ex Vivo Assay

We determined the permeability of retinal vasculature using the ex vivo Evans blue albumin method. A solution of Evans blue (E2129, Millipore Sigma, Burlington, MA, USA) (5 mg/mL dissolved in PBS pH 7.4) was introduced through an intraperitoneal injection in 3 animals per experimental group (17 mg/Kg). After injection, the animals turned blue, confirming dye uptake and distribution. After 2 h, the mice were euthanized by cervical dislocation and the eyes were enucleated. We obtained the retinas of each animal, which were flat mounted in 25.5 × 75.5 × 1.0 mm^3^ poly-L-Lysine positively charged microscopic slides (S21.2113.A, Leica Biosystems, Wetzlar, Germany) with Prolong Mounting Medium Fluorescence (P36930, Invitrogen TM, Thermo Fisher Scientific, Eugene, OR, USA) and coverslips (15747592, ThermoFisher Scientific, Waltham, MA, USA). Images were acquired from different fields at 60× using a 561 nm laser line in a confocal laser scanning microscope (Fluoview FV1000 Laser Scanning Confocal Microscope Olympus, Hamburg, Germany). All images were recorded with identical beam intensity at a size of 1024 pixels × 1024 pixels. The number of extravasations per field of 60x was counted for the quantitative analysis using Image J software (U. S. National Institutes of Health, Bethesda, MD, USA).

### 2.5. Glial Fibrillary Acidic Protein (GFAP) and DPP-4 Immunofluorescence Analysis

Paraffined ocular globes were sectioned (3 µm) and mounted on 25.5 × 75.5 × 1.0 mm^3^ poly-L-Lysine positively charged microscopic slides (S21.2113.A, Leica Biosystems, Wetzlar, Germany). Samples were deparaffinized in xylene (28975360, VWR International Eurolab, Barcelona, Spain), rehydrated in grade ethanol series (1009832500, Millipore Sigma, Burlington, MA, USA), fixed in ice-cold acid methanol (−20 °C), and washed with PBS 0.01 M at pH 7.4. Successively, slides were heated in a pressure cooker at 150 °C for 4 min in 250 mL of antigen retrieval buffer (100X citrate buffer) pH 6 diluted 1:100 (ab93678, Abcam, Cambridge, UK). Then, sections were blocked with blocking solution (10% normal goat serum (31873, ThermoFisher Scientific, Waltham, MA, USA) + 1% BSA (A3059-100G, Millipore Sigma, Burlington, MA, USA) in PBS) for 1 h at room temperature, and afterwards, they were incubated overnight at 4 °C with the anti-GFAP (rabbit monoclonal; 1:500; ab7260; Abcam, Cambridge, UK) or the anti-DPP-4 (rabbit polyclonal; 1:200; ab28340; Abcam, Cambridge, UK) primary antibodies. The next day, after three washes in PBS, sections were incubated for 1 hour in darkness with Alexa FluAE:or^®^ 488 (goat polyclonal; 1:600; ab150081; Abcam, Cambridge, UK) or Alexa Fluor^®^ 594 (goat polyclonal; 1:600; A-110012; Life Technologies, Waltham, MA, USA) secondary antibody. Sections were washed with PBS again, counterstained with Hoechst 33342 (bisbenzimide) (14533, ThermoFisher Scientific, Waltham, MA, USA), and mounted with Prolong Mounting Medium Fluorescence (P36930, InvitrogenTM, Thermo Fisher Scientific, Eugene, OR, USA) and a coverslip (15747592, ThermoFisher Scientific, Waltham, MA, USA). The images were obtained at a resolution of 1024 × 1024 pixels using laser scanning confocal microscopy (Fluoview FV1000 Laser Scanning Confocal Microscope Olympus, Hamburg, Germany) and the immunofluorescences were quantified with ImageJ software (U. S. National Institutes of Health, Bethesda, MD, USA).

### 2.6. Measurements of Glial Activation

Glial activation was evaluated by laser scanning confocal microscopy at a magnification of 60× using specific staining antibodies against GFAP. To evaluate the degree of glial activation, we used a scoring system based on the extent of GFAP staining, following the methodology described [14]. This scoring system (previously used by our group) was as follows: Müller cell endfeet region/ganglion cell layer (GCL) only (score 1); Müller cell endfeet region/GCL plus a few proximal processes (score 2); Müller cell endfeet plus many processes, but not extending to inner nuclear layer (INL) (score 3); Müller cell endfeet plus processes throughout with some in the ONL (score 4); and Müller cell endfeet plus many dark processes from GCL to the outer margin of ONL (score 5).

### 2.7. Histological Assessment with Hematoxylin-Eosin (H&E) Staining

#### 2.7.1. Cell Counting in GCL and INL

Eyes were collected and fixed in 4% paraformaldehyde (sc-281692, Santa Cruz Biotechnology, Dallas, TX, USA). After paraffin embedding, retinas were cross-sectioned (3 μm) and a hematoxylin and eosin (H&E) staining was performed for a systematic morphometric analysis of retinal layers. Images of H&E-stained sections were captured with a microscope (FSX100 Inverted Microscope Olympus, Hamburg, Germany) at a magnification of 35× and quantified using the software ImageJ (U. S. National Institutes of Health, Bethesda, MD, USA). Measurements were taken in three different regions of the central retina. Similar and comparative locations for all studied retinas were determined using the same demarcation, which consists in selecting regions close to the optic nerve but with a minimum extension of 300 µm from both sides of the optic nerve head rim (posterior pole of the eye). Cell counts of GCL and INL were averaged and compared among all the experimental groups.

#### 2.7.2. Analysis of Retinal Thickness

Total and INL thicknesses were measured using the ImageJ software (U. S. National Institutes of Health, Bethesda, MD, USA). Five distinct images of each retina were analyzed, quantified, and averaged for obtaining both thicknesses. Imaged regions were selected according to the demarcation described in the cell-counting section, and for each region, a representative value was obtained through the arithmetical mean of ten different measures.

### 2.8. Statistical Analysis

Data are expressed as the mean ± standard error of the mean (SEM). For multiple comparisons, a one-way ANOVA followed by the Bonferroni test was used. For statistical purposes, the GFAP extent score was categorized as “normal” (scores 1 and 2) and “pathological” (scores 3, 4 and 5), and for comparisons between groups, Fisher’s exact test was used. Statistical significance was set at *p* < 0.05.

## 3. Results

### 3.1. Dose–Response Effect of Topical Administration of Sitagliptin and Saxagliptin on DPP-4 Content

**Figure 1:**
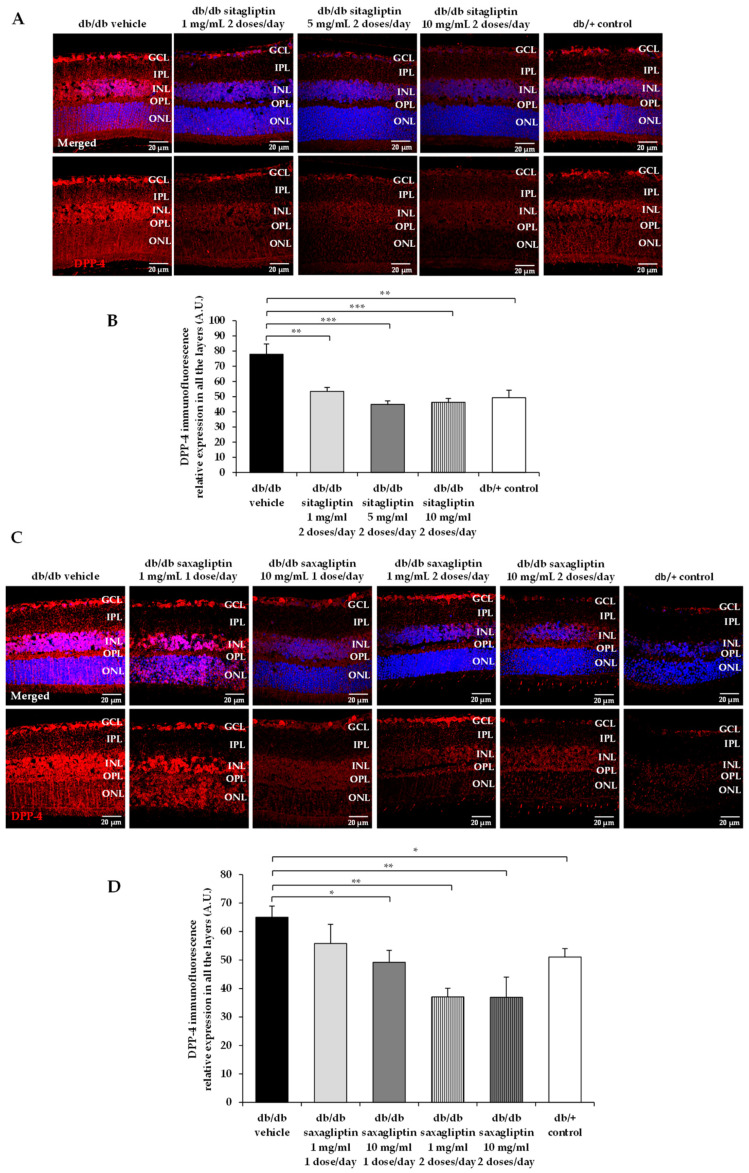
DPP-4 protein abundance. (**A**,**C**) Comparison of retinal DPP-4 immunofluorescence reactivity (red) among representative samples of each experimental group. DPP-4 relative fluorescence intensity is presented alone and merged with Hoechst nuclei staining (blue). ONL: outer nuclear layer; OPL: outer plexiform layer; INL: inner nuclear layer; IPL: inner plexiform layer; GCL: ganglion cell layer. Scale bars, 20 µm. (**B**,**D**) Quantification of the DPP-4 immunofluorescence intensity. *n* = 4 mice per group and 5 retinal sections for each retina. * *p* < 0.05; ** *p* < 0.01; *** *p* < 0.001.

Both DPP-4 inhibitors reduced the DPP-4 protein content in the diabetic retina (Figure 1). No differences were observed among sitagliptin concentrations. However, saxagliptin administered twice at day achieve a higher reduction in DPP-4 levels than when it was administered once daily.

### 3.2. Dose–Response Effect of Topical Administration of Sitagliptin and Saxagliptin on Glial Activation

**Figure 2:**
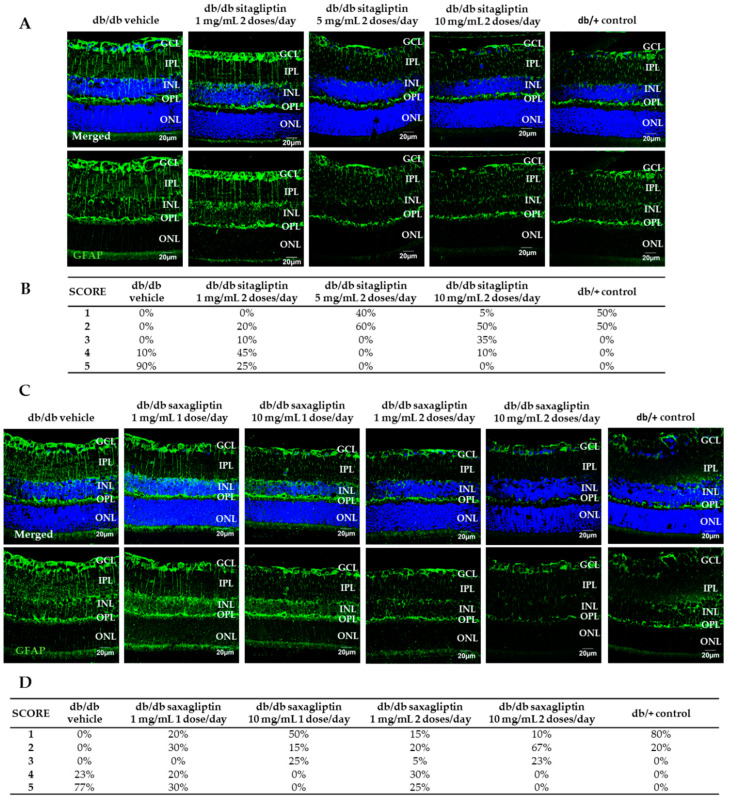
Reactive gliosis. (**A**,**C**) Comparison of GFAP immunofluorescence reactivity (green) among experimental groups to assess the dose-efficacy effect of sitagliptin (**A**), and saxagliptin (**C**). GFAP relative fluorescence intensity is displayed isolated and merged with Hoechst nuclei staining (blue). ONL: outer nuclear layer; OPL: outer plexiform layer; INL: inner nuclear layer; IPL: inner plexiform layer; GCL: ganglion cell layer. Scale bars, 20 μm. (**B**,**D**) Quantification of reactive gliosis based on the extent of GFAP staining. *n* = 4 mice per group and 5 retinal sections for each retina.

Non-diabetic mice exhibited GFAP scores < 3 (Figure 2A–D), whereas vehicle-treated diabetic mice displayed a minimum score of four as a result of the aberrant expression of GFAP (Figure 2A–D). Sitagliptin (with the exception of the lowest concentration, 1 mg/mL) prevented reactive gliosis, 5 mg/mL being the more effective concentration (Figure 2A,B). Regarding saxagliptin, the higher concentration (10 mg/mL) produced the higher reduction in glial activation (Figure 2C,D), regardless if it was administered once or twice per day.

### 3.3. Dose–Response Effect of Sitagliptin and Saxagliptin Eye Drops on Cell Death and Retinal Thinning

**Figure 3:**
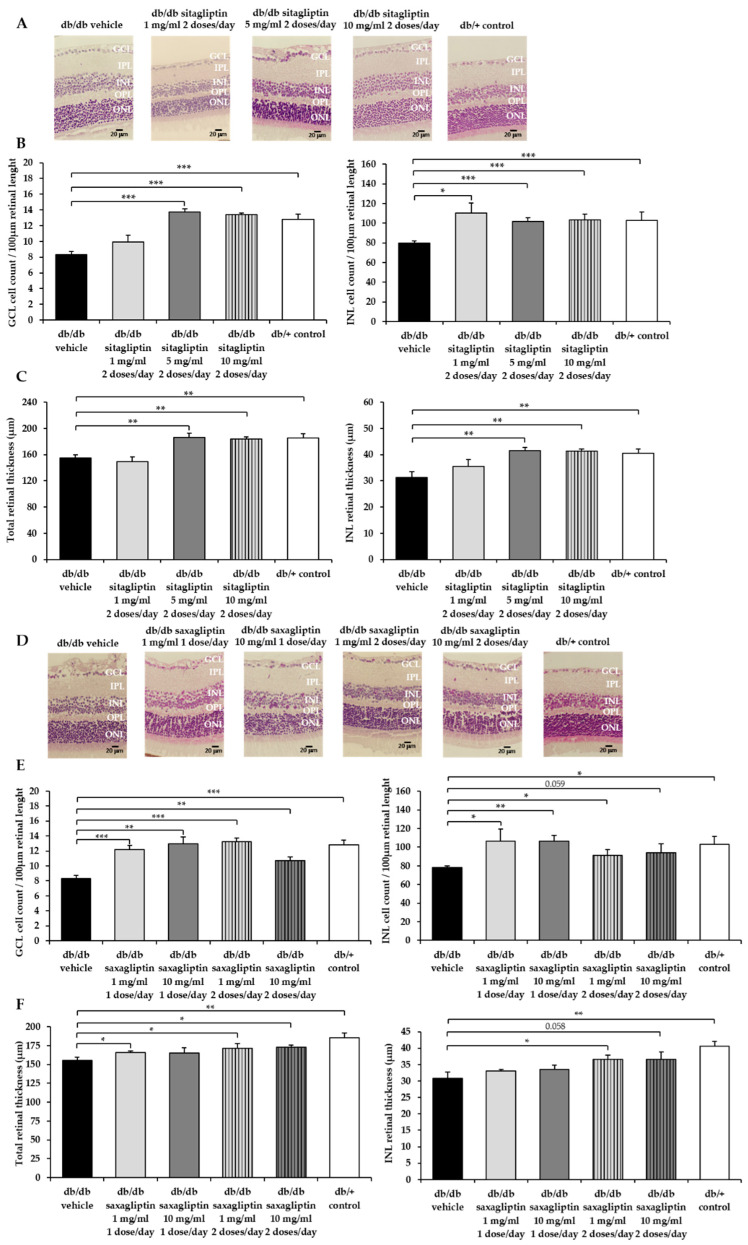
Cell count and retinal thinning. (**A**,**D**) Comparison of hematoxylin/eosin (HE)-stained retinas among representative samples of each experimental group. ONL: outer nuclear layer; OPL: outer plexiform layer; INL: inner nuclear layer; IPL: inner plexiform layer; GCL: ganglion cell layer. Scale bars, 20 µm. (**B**,**E**) Quantification of the number of cells in the GCL and INL layers. (**C**,**F**) Retinal thickness of the INL layer and the whole retina. *n* = 4 mice per group and five retinal sections for each mouse. * *p* < 0.05; ** *p* < 0.01; *** *p* < 0.001.

Diabetic mice treated with vehicle showed a clear reduction in the GCL and INL cell counts and in the retinal thickness measurements in comparison to control mice (Figure 3A–F).

Topical administered sitagliptin at concentrations of 5 mg/mL and 10 mg/mL were able to promote cell survival and to preserve retinal thinning, while the results obtained with 1 mg/mL were similar to those obtained in diabetic animals treated with vehicle (Figure 3A–C). Regarding saxagliptin, both concentrations reduced neuronal cell death in the GCL and INL, but only when it was administered twice daily did it result in a significant impact on the INL and total retinal thicknesses.

### 3.4. Dose–Response Effect of Topical Administration of Sitagliptin and Saxagliptin on Vascular Leackage

**Figure 4:**
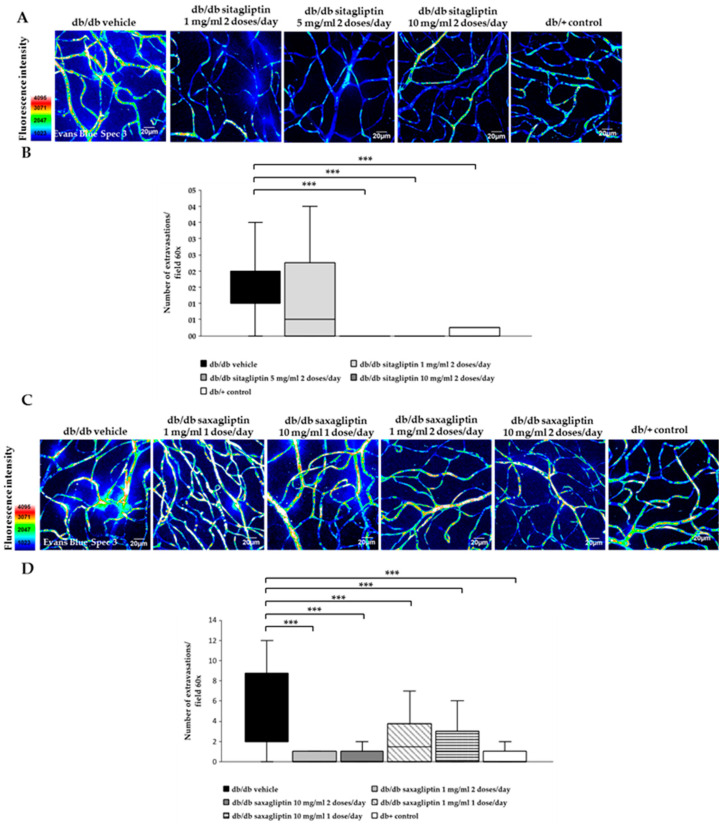
Microvascular abnormalities. (**A**,**C**) Confocal images of vascular permeability assessed by Evans blue dye leakage in retinal whole mounts. Treatments with sitagliptin are compared in panel (**A**) and saxagliptin treatments in panel (**C**). Spec3, fluorescent spectral signature 3. Scale bars, 20 μm. (**B**,**D**) Quantification of extravasations per 60× field of the retina. Treatments with sitagliptin are compared in panel (**B**) with saxagliptin treatments in panel (**D**). In graph (**B**) vehicle is presented with black bars, sitagliptin 5 mg/mL 2 doses/day with grey bars, sitagliptin 10 mg/mL 2 doses/day with dark grey bars and control mice with white bars. In graph (**D**) vehicle is presented with black bars, saxagliptin 1 mg/mL 1 dose/day with white bars with diagonal lines, saxagliptin 10 mg/mL 1 dose/day with white bars with horizontal lines, saxagliptin 1 mg/mL 2 doses/day with grey bars, saxagliptin 10 mg/mL 2 doses/day with dark grey bars and control mice with white bars. *n* = 3 mice per group, 25–30 fields per animal were analyzed. *** *p* < 0.001.

As a consequence of multiple extravasations points, higher Evans blue fluorescence was observed in blood vessels in diabetic mice treated with vehicle in comparison to control mice (Figure 4A,C). The higher concentrations of sitagliptin (5 and 10 mg/mL) clearly ameliorated this vascular abnormality, while 1 mg/mL did not prevent vascular leakage (Figure 4A,B). Saxagliptin was also able to prevent vascular leakage, being more effective when administered twice per day, independently of the concentration (Figure 4C,D).

## 4. Discussion

In the present study, we evaluated the lower effective dose of a topical (eye drops) administration of sitagliptin and saxagliptin for preventing the hallmarks of NVU impairment: reactive gliosis, neural apoptosis, and vascular leakage. We found that 5 mg/mL for sitagliptin, and 10 mg/mL for saxagliptin, both administered twice daily, were the minimum effective doses to prevent all these retinal abnormalities, which are essential in the early stages of experimental DR.

Whereas sitagliptin inhibits DPP-4 enzyme by forming non-covalent bonds with its catalytic site, saxagliptin forms a covalent enzyme–inhibitor complex with slow rates of dissociation and consequently longer durations of action [15]. For this reason, we wanted to examine whether saxagliptin could be administered once per day instead of twice per day. We found that whereas glial activation was abrogated when using saxagliptin once per day, neuroretinal thinning and vascular leakage were not significantly reduced. Therefore, as occurs with sitagliptin, saxagliptin should be administered twice per day to obtain full neurovascular protection.

We observed that sitagliptin and saxagliptin downregulated the DPP-4 content in the diabetic retina. Although we do not have any reliable explanation for this finding, it would be related to the reported GLP-1 enhancement that occurs after topical ocular administration of DPP-4 inhibitors in the same experimental model [13].

Neuroprotection is considered an essential part of the treatment of the early stages of DR. Recently, the term DRD instead of DR has been proposed to emphasize the concept that DR is not only a microvascular disease [16]. In this regard, the American Diabetes Association currently defines DR as a specific neurovascular disease [17]. In this setting, topical ocular administration of neuroprotective agents has emerged as a new strategy for treating the early stages of DR due to several reasons: (1) It is a non-invasive route that permits self-application by the patient. (2) There is evidence that a lot of drugs are able to reach the retina at pharmacological concentrations, and multiple experimental studies have demonstrated the effectiveness of eye drops for treating the early stages of DR in experimental models [18]. (3) The systemic side effects are significantly lower than when using systemic administration. (4) The blood–retinal barrier, a serious limiting factor when trying to reach the retina when systemic administration is used, can be sorted out by a topical ocular route.

A topical (eye drops) administration of DPP-4 inhibitors not only exerts a powerful neuroprotective action but also prevent vascular leakage and, therefore, these inhibitors can be envisaged as excellent candidates for treating the early stages of DR. The rationale for using eye drops containing DPP-4 inhibitors is based on the presence of GLP-1 and DPP-4 in the retinas of mice and humans [7,13]. GLP-1 is expressed mainly in the ganglion cell layer and is downregulated in diabetes [7]. In addition, the retinal pigment epithelium of subjects with diabetes present higher DPP-4 concentrations than non-diabetic controls matched by age [13]. Therefore, the already decreased amount of GLP-1 in the diabetic retina can be even worsened by its exaggerated degradation induced by DPP-4. Consequently, the replacement of GLP-1 in the retina can be considered a target for treating DR. This can be achieved by administering GLP-1, by preventing its degradation or by combining both strategies. The advantage of using DPP-4 inhibitors is that they are cheaper, more stable than GLP-1, and can exert other pleiotropic actions unrelated to GLP-1, which are always welcome in a complex and multi-pathway disease such as DR. In this regard, Dietrich et al. [19] have shown that the DPP-4 linagliptin has a neuroprotective effect in *Caenorhabditis elegans*, a model of hyperglycemia-induced neurodegeneration in which GLP-1R is not produced. It could be argued that given than DPP-4 is a well-established oral treatment for type 2 diabetes, it would be unnecessary to use the topical (eye drops) administration, at least in patients undergoing DPP-4 treatment. However, current evidence does not indicate any beneficial effect of the systemic administration of DDP-4 inhibitors [20,21] on DR. This could be due to their inability to cross the blood–retinal barrier (BRB). Since DPP-4 inhibitors are unable to cross the blood–brain barrier [22,23], it is broadly assumed that they do not cross the BRB.

In conclusion, in the present study the minimum effective doses of sitagliptin and saxagliptin, administered by eye drops, for treating the early stages of DR have been established in the db/db mouse model. These data can be used for the design not only for preclinical regulatory purposes but also for planning future clinical trials.

## Data Availability

The data presented in this study are available on request from the corresponding author.
